# Serum depletion induces changes in protein expression in the trophoblast-derived cell line HTR-8/SVneo

**DOI:** 10.1186/s11658-016-0018-9

**Published:** 2016-10-16

**Authors:** Susana Novoa-Herran, Adriana Umaña-Perez, Francesc Canals, Myriam Sanchez-Gomez

**Affiliations:** 1 Departamento de Química, Grupo de Investigación en Hormonas (Hormone Research Laboratory), Universidad Nacional de Colombia, Sede Bogotá, Facultad de Ciencias, Cra 30 45-03 Ed 451 Of 464, AA 111321 Bogotá, Colombia; 2 Laboratory of Proteomics, Vall d’Hebron Institute of Oncology (VHIO), Centre Cellex, C Natzaret 115-117, 08035 Barcelona, Spain

**Keywords:** Cancer, Metabolism, Proteomics, Placenta, Cell culture, Serum, Vimentin, MTT, Trophoblast, Western blot

## Abstract

**Background:**

How nutrition and growth factor restriction due to serum depletion affect trophoblast function remains poorly understood. We performed a proteomic differential study of the effects of serum depletion on a first trimester human immortalized trophoblast cell line.

**Methods:**

The viability of HTR-8/SVneo trophoblast cells in culture with 0, 0.5 and 10 % fetal bovine serum (FBS) were assayed via MTT at 24, 48 and 64 h. A comparative proteomic analysis of the cells grown with those FBS levels for 24 h was performed using two-dimensional electrophoresis (2DE), followed by mass spectrometry for protein spot identification, and a database search and bioinformatics analysis of the expressed proteins. Differential spots were identified using the Kolmogorov-Smirnov test (*n* = 3, significance level 0.10, D > 0.642) and/or ANOVA (*n* = 3, *p* < 0.05).

**Results:**

The results showed that low serum doses or serum depletion differentially affect cell growth and protein expression. Differential expression was seen in 25 % of the protein spots grown with 0.5 % FBS and in 84 % of those grown with 0 % FBS, using 10 % serum as the physiological control. In 0.5 % FBS, this difference was related with biological processes typically affected by the serum, such as cell cycle, regulation of apoptosis and proliferation. In addition to these changes, in the serum-depleted proteome we observed downregulation of keratin 8, and upregulation of vimentin, the glycolytic enzymes enolase and pyruvate kinase (PKM2) and tumor progression-related inosine-5’-monophosphate dehydrogenase 2 (IMPDH2) enzyme. The proteins regulated by total serum depletion, but not affected by growth in 0.5 % serum, are members of the glycolytic and nucleotide metabolic pathways and the epithelial-to-mesenchymal transition (EMT), suggesting an adaptive switch characteristic of malignant cells.

**Conclusions:**

This comparative proteomic analysis and the identified proteins are the first evidence of a protein expression response to serum depletion in a trophoblast cell model. Our results show that serum depletion induces specific changes in protein expression concordant with main cell metabolic adaptations and EMT, resembling the progression to a malignant phenotype.

**Electronic supplementary material:**

The online version of this article (doi:10.1186/s11658-016-0018-9) contains supplementary material, which is available to authorized users.

## Background

The human placenta is composed of trophoblast cells. The correct development of the embryo depends on trophoblast function. These cells invade the uterus and remodel the maternal spiral arteries. The intrauterine environment of the first trimester plays an important role in the regulation of trophoblast function and capacities [1]. Trophoblast cells share several skills with cancer cells, leading to the descriptions such as “pseudo-malignant” or “physiological metastasis” despite their spatial and temporal regulation [2].

Several trophoblast cell lines have been developed to study trophoblast function in vitro. One example is the immortalized extravillous trophoblast HTR-8/SVneo cell line, which was originally obtained from a first trimester human placenta.

Fetal bovine serum (FBS) is traditionally used to simulate human physiological conditions because it contains many of the appropriate nutrients and signal molecules. Although its composition differs from human serum, human cells can respond to bovine proteins thanks to their homology. FBS is used as a supplement for cell culture media, allowing cell growth, and varying its dose can be used to simulate pathological or treatment conditions.

Serum depletion can influence the radiation-induced killing of pancreatic cancer cells [3], allow the dexamethasone-mediated differentiation of neuroblastoma cells [4], and give rise to growth inhibition, differentiation and interleukin 1 receptor expression in leukemia cells [5]. However, the effect of serum depletion on trophoblast function remains unknown.

The tumor microenvironment influences the progression of cancer [6] similarly to how the intrauterine environment influences placental function [1]. It is known that FBS can stimulate the proliferation, migration and invasion of HTR-8/SVneo trophoblast cells in vitro [7–9], although the protein expression profile under these conditions remains to be examined. In this study, we investigated the effect of serum depletion on trophoblast protein expression and profile with the aim of determining the impact on trophoblast survival. Designating 10 % FBS as the control level (the model of physiological growth) and 0.5 or 0 % FBS as partially or completely depleted states, we conducted two proteomic studies using immortalized human HTR-8/SVneo trophoblastic cells. The proteomes were separated via two-dimensional gel electrophoresis (2DE), and protein spots were identified via tandem mass spectrometry (MS/MS) and enrichment annotation and protein interaction network analyses. Understanding the profile of proteins that are differentially expressed serum depletion occurs may generate important information on the relationship between trophoblast cells and nutrient availability, environment and functionality.

## Methods

### Cell culture conditions

Dr. Angela Cadavid of the Universidad de Antioquia in Colombia provided the HTR-8/SVneo cell line. This cell line was developed from an explant culture of a human first trimester extravillous trophoblast and immortalized [7]. Our group has performed additional studies and characterization assays [10–12]. Cells were grown in a monolayer at 37 °C in a humidified atmosphere with 5 % CO_2_ in RPMI 1640 medium (Sigma Chemical Co.) supplemented with 10 % FBS, 200 mM L-Glutamine and antibiotics (100 Units/ml of penicillin/100 μg/ml streptomycin; Thermo Fischer Scientific). 1 x 10^6^ cells were plated in triplicate in 100 mm tissue culture dishes for two days with 7 ml of growth medium/10 % FBS, starved overnight in serum-free medium and subsequently incubated for 24 h with 0, 0.5 or 10 % FBS.

### MTT assay

Cell proliferation was evaluated based on the metabolic activity in viable cells using a 3-(4,5-dimethylthiazol-2-yl)-2, 5-diphenyl tetrazolium bromide (MTT) assay (Sigma-Aldrich Co.) [13]. For this assay, HTR-8/SVneo cells were plated in triplicate in a 96-well flat-bottom tissue culture plate at a concentration of 1 x 10^4^ cells/well containing 200 μl of RPMI 1640 medium (Sigma Chemical Co.) supplemented with 10 % FBS (Thermo Fischer Scientific). Cells that had attached by 12 h were starved overnight in serum-free medium and subsequently cultured with 0, 0.1, 0.5 or 10 % FBS for 0, 24, 48 or 64 h. At each time, 10 μl of 5 mg/ml MTT solution was added per well and incubated for 4 h. The culture medium was then removed and formazan-generated crystals were dissolved with 100 μl of dimethylsulfoxide (DMSO, Sigma Chemical Co.). The plate was shaken for 3 s in a BioRad Microplate Reader 680 and the absorbance was read in dual reading mode with the measurement filter at 570 nm and reference filter at 630 nm. Changes in cell number were confirmed and monitored via contrast-phase microscopy (Leica DMIL).

### General sample preparation

Whole cell lysis was done in RIPA buffer, as previously described [14]. The extract was cleaned with chloroform/methanol and redissolved in 500 μl IEF sample buffer consisting of 7 M urea, 2M thiourea, 4 % CHAPS, 40 mM dithiothreitol (DTT), and 1 % ampholites (pH 3–10). Protein quantitation was done using the Pierce 660 nm protein kit (Thermo Fischer Scientific) with bovine serum albumin as the protein standard.

### Protein separation by fluorescent 2-D difference gel electrophoresis, image acquisition and analysis

The protein extract was further cleaned using a modified trichloroacetic acid–acetone precipitation method (2-D-CleanUp kit, GE Healthcare), and dissolved in DIGE labeling buffer to a final concentration around 5 μg/μl. The exact protein concentration was determined using the BioRad RCDC Protein Assay.

The protein in each sample was labeled with N-hydroxy succinimidyl ester derivatives of the cyanine dyes Cy2, Cy3 and Cy5 and separated using fluorescent 2-D difference gel electrophoresis (DIGE) as described previously [15]. Forty μg of protein in each lysate were labeled with 400 pmol of either Cy2 or Cy3 for comparison on the same 2D gel.

A pool of all samples was also equitably prepared and 40 μg were labeled with Cy5 to be used as the internal standard on all gels to aid image matching and cross-gel statistical analysis. Thirty μg of an invisible pool of all samples was included to aid in MS identification.

The Cy2 and Cy3 labeling reactions from each lysate were mixed and run on the same gels with the Cy5-labeled standard and invisible pool. First dimension isoelectric focusing (IEF) electrophoresis was performed using immobilized pH gradient strips (24 cm, pH 3–10 nonlinear gradient) on an Ettan Immobilized pH Gradientphor System. Strips were passively rehydrated overnight with 450 μl of rehydration buffer consisting of 7 M urea, 2 M thiourea, 4 % wt/vol CHAPS, 1 % pharmalytes (pH 3–10), 100 mM DeStreak and 0.002 % bromophenol blue. Samples were applied via cup loading near the acidic end of the strips. IEF was performed at 20 °C until reaching a global voltage of 67 kV. Strips were then reduced in equilibration buffer with 5 mg/mL DTT, followed by alkylation in equilibration buffer with 22.5 mg/ml iodoacetamide, for 15 min each one and gentle shaking.

Second-dimension SDS–polyacrylamide gel electrophoresis (SDS-PAGE) was carried out on 12 % polyacrylamide gels (24 × 20 cm) cast in low fluorescence glass plates on an Ettan DALTsix system (GE Healthcare). The gels were run at 20 °C at a constant power of 2.5 W per gel for 30 min followed by 17 W per gel until the bromophenol blue tracking front reached the end of the gel. Fluorescence images of the gels were acquired on a Typhoon 9400 scanner (GE Healthcare).

Cy2, Cy3 and Cy5 images were respectively scanned at excitation/emission wavelengths of 488/520, 532/580 and 633/670 nm at a 100 μm resolution. The image analysis and statistical quantification of relative protein abundances were performed using Progenesis SameSpots software (Nonlinear Dynamics). The gels were stained with Flamingo Fluorescent Gel Stain (BioRad) and the selected protein spots were picked out with an Ettan Spot Picker (GE Healthcare).

### Protein separation by 2DE, image acquisition and analysis

Two-dimensional gel electrophoresis (2DE) was done as previously described [14]. Briefly, IEF electrophoresis was performed using immobilized pH gradient strips (18 cm, pH 3–10 nonlinear; BioRad) loaded with 0.50 mg protein per sample adjusted to 300 μl with rehydration buffer (urea/thiourea/CHAPS/DTT). The strips were passively rehydrated for 2 h at room temperature, followed by active rehydration for 11 h at 50 V on a Protean IEF System (BioRad). IEF was performed at 20 °C using the following protocol: 250 V for 30 min, 1000 V for 30 min, 1000 V for 1 h (constant), 4000 V for 45 min, 4000 V for 1 h (constant) and 8000 V until 55 kVh was reached.

The strips were then reduced in equilibration buffer with 130 mM DTT, followed by alkylation in equilibration buffer with 135 mM iodoacetamide, for 20 min each with gentle shaking. Second dimension SDS-PAGE was carried out on a 12 % polyacrylamide gel in the DODECA Electrophoresis System (BioRad) at a constant voltage of 80 V for 10 h. Gels were stained with Colloidal Coomassie staining based on a modified Peisker protocol [14, 16]. The documented gels were analyzed with ImageMaster 2D Platinum 7.02 software (GE Healthcare).

### Statistical analysis

Two-way analysis of variance (ANOVA) followed by Bonferroni post-hoc test (*n* = 3, *p* < 0.05) was used to compare the metabolic activity differences revealed in the MTT assay. The data are expressed as mean and standard deviation. For 2DE and DIGE proteomic analysis, three independent experiments were used. Each one of three culture dishes for each condition were separated on 2DE gels and analyzed. Protein spot comparison was done according to the software manufacturer’s recommendation, and differentially expressed proteins in the gels were identified using the Kolmogorov-Smirnov test (*n* = 3, significance level 0.10, D > 0.642 parameter) and/or ANOVA test (*n* = 3, *p* < 0.05).

### Protein identification via mass spectrometry (comparative profile)

In-gel trypsin digestion was performed using autolysis-stabilized trypsin (Promega). Protein spots of DIGE gels were analyzed using matrix-assisted laser desorption/ionization–mass spectrometry analysis (MALDI-MS), while protein spots of triplicate 2DE gels were analyzed using liquid chromatography electrospray–mass spectrometry analysis (LC-ESI-MS/MS).

MALDI-MS of tryptic peptides was performed on an Ultraflex TOF/TOF mass spectrometer (Bruker). Samples were prepared using α-cyano-4-hydroxy-cinnamic acid as matrix on anchor-chip targets (Bruker). Identification of the proteins was carried out via peptide mass fingerprinting data and/or TOF/TOF post source decay fragmentation spectra. Database searches were performed using the MASCOT program (Matrix Science).

LC-ESI-MS/MS analysis of tryptic peptides was performed on an Agilent Technologies Q-TOF LC-ESI-MS/MS Instrument equipped with 1200 Series liquid chromatography, chip cube ESI MS interface, and 6530 Q-TOF mass spectrometer. The instrument was operated via the MassHunter Data Acquisitions software. All MS/MS were processed using qualitative analysis software. Peptide identification was carried out using Mascot Daemon 2.1.6. Searches were performed against the IPI human database (build 3.70). Trypsin was used as the enzyme and one missed cleavage was allowed. Carbamidomethylation (CAM) for cysteine was set as the fixed modification and oxidized methionine as the variable modification. Tolerance for precursor masses was set to 50 ppm for PMF and to 0.5 Da for fragment ions in TOF/TOF spectra. The criteria for positive identification were a Mascot protein score greater than the significance threshold (*p* < 0.05) for PMF, and an ion score greater than the Mascot identity threshold for MS/MS matches.

### Bioinformatics analysis: annotation of identified proteins

Gene ontology (GO) annotation for biological processes, molecular functions and related pathways was done using Nextprot (release 2014-09-19, SIB & GeneBio; http://www.nextprot.org) and DAVID Functional Annotation Tool (DAVID Bioinformatics Resources 6.7, National Institute of Allergy and Infectious Diseases, NIH; http://david.ncifcrf.gov). Enrichment annotation analyses of identified proteins were performed using DAVID. Functional annotation clustering analysis was used with *Homo sapiens* as the background, a medium classification stringency level, and using PANTHER GO terms and the entire available pathways database as well as default annotations. Terms with p < 0.05 were selected and each generated cluster was named according to a representative term of molecular function, biological process and pathway, including the overall term. Analysis of direct (physical) and indirect (functional) associations among the identified proteins was done using STRING 9.05 software [17]. A confidence view was used with a medium confidence score (0.400), no additional node and all the prediction methods active. Biological relevance was assessed using PCViz software [18]. All of the identified proteins except the hub protein ACTG were used as input, and the background nodes were added until the size set was 4 time larger.

### Western blot analysis

Differences in vimentin expression between samples were assayed using anti-vimentin rabbit monoclonal antibody (EPR3776, Abcam) and anti-rabbit IgG HRP-conjugated antibody (NA 934, Amersham Biosciences) at 1:5,000 or 1:10,000 dilution in TBS, respectively. 30 μg of whole-cell protein extracts from each treatment were separated using SDS-PAGE and transferred to nitrocellulose membranes by wet electroblotting, as described previously [14]. Non-specific binding sites were blocked with 5 % fat-free milk powder in TBS (pH 7.4) for 3 h. The membranes were incubated with 1:5,000 TBS-dilution of primary anti-vimentin rabbit monoclonal antibody (EPR3776, Abcam) overnight at 4 °C, then washed with TBS for 10 min three times, incubated with 1:10,000 TBS dilution of secondary anti-rabbit IgG HRP-conjugated antibody (NA 934, Amersham Biosciences) for 2 h at room temperature, and washed with TBS for 10 min three times.

Signals were visualized using the ECL chemiluminescence system (ab5801, Abcam) and detected on film (CL-XPosure Film, Thermo Scientific) using the exposition time that generated the best signal. Protein molecular weight was determined using PageRuler Plus Prestained Protein Ladder (Thermo Scientific) and assigned using Quantity One software 4.6. (BioRad). Densitometric quantifications were performed in triplicate using Quantity One software.

## Results

### The effects of FBS on trophoblast cell growth are dose dependent

**Fig. 1:**
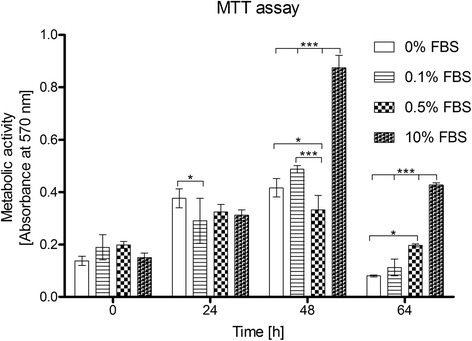
Viability and growth of HTR-8/SVneo cells culture at variable serum doses. Effect of 0, 0.1, 0.5 and 10 % FBS doses on the growth of HTR-8/SVneo cells assayed via MTT at 0, 24, 48 and 64 h. Two-way ANOVA test and Bonferroni post-test were applied (*n* = 3, *p < 0.05, ***p < 0.001)

Using the MTT assay, we determined that HTR-8/SVneo cells have a high metabolic activity and proliferate both in the presence or absence of FBS. The spectrophotometry results correlated with an increase in cell number that could be monitored via contrast-phase microscopy. As shown in Fig. 1, 0.5 % FBS plays a support role without significantly affecting cell growth. At lower FBS doses, HTR-8/SVneo cells behave similarly to what is seen in serum-depleted conditions (not significant difference *p* > 0.005 at 48 and 64 h), with a significantly higher proliferation rate compared to 0.5 % FBS culture at 48 h (*p* < 0.05 to 0 % FBS; *p* < 0.001 to 0.1 % FBS).

Cell viability with 0 % and 0.1 % FBS suffers a significantly strong fall at 64 h compared to the viability at 0.5 % FBS (*p* < 0.05 to 0 % FBS). Cell number was verified optically, determining a higher cell number in 0, 0.1 and 10 % FBS cell cultures compared to that for the 0.5 % FBS culture at 24 and 48 h. These results denote an additional effect of serum depletion over cell growth, compared with low serum doses as 0.5 %. Thus, serum depletion could not only affect metabolic processes, but may also influence the protein expression profile.

### 0.5 % serum level slightly affects trophoblast protein expression

**Fig. 2:**
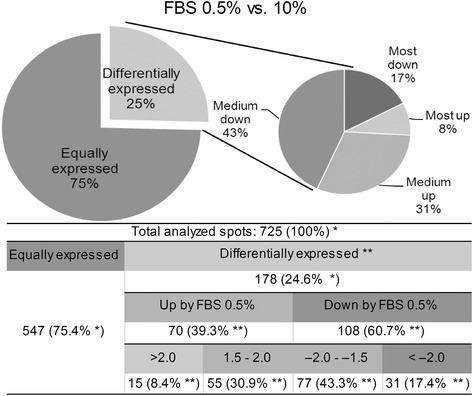
Comparative proteomic analysis of proteins expressed by HTR-8/SVneo cells cultured with variable serum doses. Percentage of differentially expressed protein spots in HTR-8/SVneo trophoblast cells cultured in 0.5 or 10 % FBS for 24 h. *n* = 3, ANOVA test, *p* < 0.05. * –percentage of total analyzed spots; ** – percentage of differentially expressed spots

We first performed a proteomic assay using 7-cm, pI 4–7, 2DE gels; differential and invariable spots in comparison with control (10 % FBS culture) were selected and identified via MALDI-MS, yielding up to 34 unique identified proteins (Additional file 1). Then, using the DIGE strategy, we compared the proteomic profiles of HTR-8/SVneo cells grown with 0.5 and 10 % FBS for 24 h. Results showed that 25 % of protein spots were differentially expressed, out of which 39.3 % were upregulated and 60.7 % were downregulated by 0.5 % FBS. In general, the main changes were moderate (1.5- to 2.0-fold in 30.9 % cases; –2.0- to –1.5-fold in 43.3 % cases; Fig. 2). 120 protein spots were selected among differential and invariable (–1.5- to 1.5-fold) spots, and identified via MALDI-MS, yielding 69 unique identified proteins (Additional file 2). The identified proteins were classified with GO annotations for biological process, molecular functions and pathways. Interestingly, among the proteins downregulated by 0.5 % serum, there are proteins lined to cell differentiation, key regulators of chromosome segregation during the cell cycle, and cytoskeleton-dependent intracellular transport proteins. Detailed protein information for the comparative proteomic analysis is provided in Additional file 1: Table S2 and Additional file 2: Table S3.

**Fig. 3:**
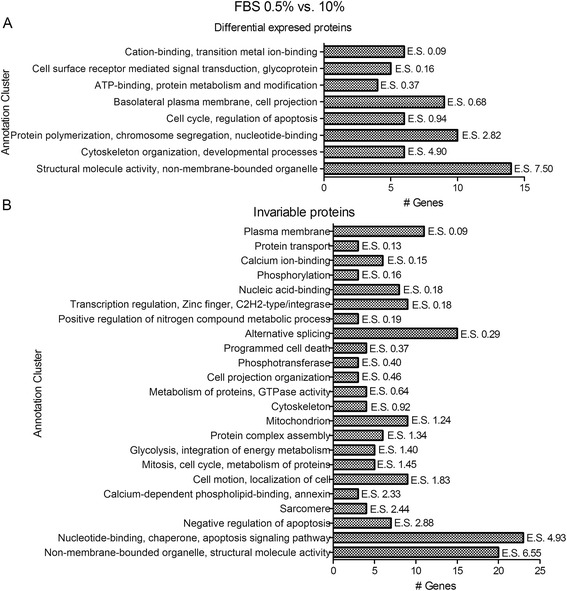
Comparative proteomic analysis of proteins expressed by HTR-8/SVneo cells cultured with variable serum doses. Enrichment annotation analyses for differential (**a**) and invariable (**b**) proteins identified are shown. Functional annotation clustering was performed with DAVID. E.S. – enriched score of functional annotation clustering

We performed a detailed enrichment annotation analysis for the differential and invariable protein groups. Differentially expressed proteins were grouped with terms for cell cycle (BP00282: mitosis p:2.0 × 10^-3^; GO:0007049 ~ cell cycle, p:4.6 × 10^-3^), regulation of apoptosis (GO:0042981 ~ regulation of apoptosis, p:0.335), chromosome segregation (BP00206: chromosome segregation p:6.5 × 10^-4^), cytoskeleton organization (GO:0007010 ~ cytoskeleton organization p:2.5 × 10^-2^) and other biological processes and molecular functions, concordant with the physiological role of the serum (Fig. 3a).

By contrast, the invariable group included proteins related to glycolytic metabolism (GO:0006096 ~ glycolysis p:1.0 × 10^-2^; hsa00010: glycolysis/gluconeogenesis p:2.7 × 10^-2^; Fig. 3b).

### Serum depletion affects trophoblast protein expression

**Fig. 4:**
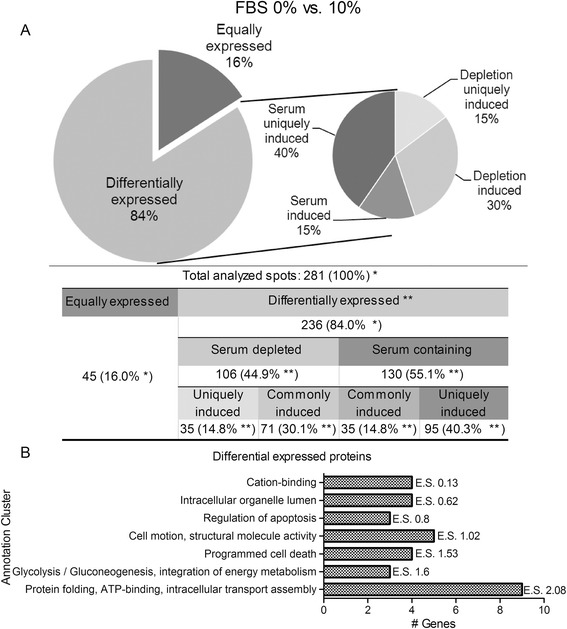
Comparative proteomic analysis of proteins expressed by HTR-8/SVneo cells cultured under serum-depleted conditions. **a** Spot percentage of differentially expressed protein spots in HTR-8/SVneo trophoblast cells cultured in 0 or 10 % FBS for 24 h. *n* = 3, Kolmogorov-Smirnov test, 0.10, D > 0.642; ANOVA test, *p* < 0.05. * – percentage of total analyzed spots; ** – percentage of differentially expressed spots. **b** Enrichment annotation analyses for differential proteins identified. Functional annotation clustering was performed with DAVID. E.S. – enriched score of functional annotation clustering

Next, we investigated the influence of serum depletion on the profile of expressed proteins through a comparative 2DE analysis with three biological replicates. Our results indicated that 84 % of the 281 analyzed protein spots showed a statistically significant differential expression, of which 44.9 % were induced in absence of FBS. Of these differentially expressed spots, 40.3 % were observed exclusively in the presence of serum, whereas 14.8 % were only present in the absence of serum (Fig. 4a).

**Fig. 5:**
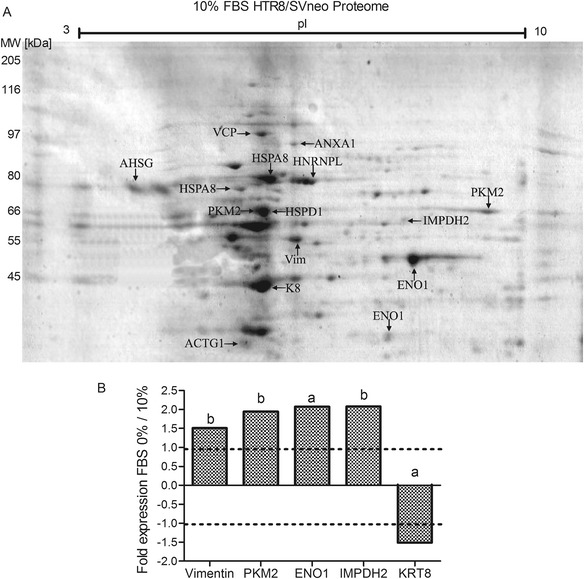
Proteins differentially expressed by HTR-8/SVneo cells cultured under serum-depleted conditions. **a** HTR-8/SVneo cells were grown in medium with 10 or 0 % FBS for 24 h (in triplicate). Total protein extracts (0.50 mg) were separated by 18 cm 2DE gels as previously described (*n* = 3). The protein spots were analyzed via LC ESI-Q/TOF and identified using bioinformatics analysis. MW – molecular weight marker (RPN5800, GE). **b** Effect of serum depletion on the expression of some trophoblast proteins. Statistical analysis of 2DE protein spots identified by MS/MS in HTR-8/SVneo proteomes either with 10 % FBS or without FBS (*n* = 3). Kolmogorov-Smirnov test (D value): 0.10, D > 0.642 (a, D = 1.0; b, D = 0.67)

Differential protein spots were identified via LC-ESI-MS-Q/TOF analysis followed by a MASCOT database search. We identified 15 protein spots corresponding to 13 unique proteins (Table 1). Results show that serum depletion had a regulatory role on protein expression. Interestingly, and in contrast to the preceding analysis for 0.5 % serum cultures, we found that proteins with important roles in metabolism and phenotype regulation were induced by serum depletion, such as vimentin (VIM), M1/M2 pyruvate kinase (PKM1/2), alpha enolase (ENO1) and inosin (IMPDH2). The expression of cytokeratine 8 (KRT8) was repressed by serum depletion (Fig. 5 and Additional file 3).

**Table 1:** Relevant proteins identified via MS/MS and bioinformatics analysis for the effects of serum depletion

Gene name	Protein name	MW (Da)	pI~	MS/MS score	% Seq	Pep match	K test	Fold FBS 0%/10%	Molecular function	Biological process
ACTG1	Actin, cytoplasmic 2	41793	5.31	221.9	47	15	1.00	^a^	Structural constituent of cytoskeleton	Cellular component movement
VCP	Transitional endoplasmic reticulum ATPase	89322	5.14	350.8	39	25	1.00	^a^	Hydrolase	DNA repair, vesicle transport
ANXA1	Annexin A1	38714	6.64	98.6	17	48	1.00	^a^	Helicase activity, Receptor binding	Cell surface receptor signaling pathway
HSP90AA1	Heat shock protein HSP 90-alpha	84660	4.94	37.67	4	3	1.00	^a^	Chaperone	Stress response
HSPA8	Heat shock cognate 71 kDa protein	70898	5.37	562.7	68	36	1.00	-3.14	Chaperone, repressor	Stress response, transcription
HNRNPL	Heterogeneous nuclear ribonucleoprotein L	64133		47.04	6	4	1.00	-2.75	Transcription regulatory region DNA binding.	RNA processing
KRT8	Keratin, type II cytoskeletal 8	53705	5.52	261.4	41	21	1.00	-1.51	Structural constituent of cytoskeleton, structural molecule activity	MET, cytoskeleton organization
HSPD1	60 kDa heat shock protein, mitochondrial	61055	5.7	497.2	66	34	0.67	-1.35	Chaperone	Host–virus interaction
VIM	Vimentin	53652	5.06	332.7	47	23	0.67	1.51	Structural constituent of cytoskeleton	EMT
DLD	Dihydrolipoyl dehydrogenase, mitochondrial	54150	6.92	54.93	9	4	1.00	1.66	Dihydrolipoyl dehydrogenase activity	Cell redox homeostasis, lysine catabolic process
PKM2	Pyruvate kinase isozymes M1/M2	57937	7.96	329.1	44	22	1.00	1.94	Kinase, transferase	Glycolysis
IMPDH2	Inosine-5’-monophosphate dehydrogenase 2	55805	6.44	83.48	13	6	0.67	2.08	Oxidoreductase	GMP and purine biosynthesis
ENO1	Alpha-enolase	47169	6.99	142.5	26	11	0.67	2.07	Lyase, repressor	Glycolysis, transcription regulation

Protein spots were analyzed via LC-ESI-MS-Q/TOF and identified using the MASCOT search engine. MW (Da) – theoretical molecular weight in Dalton; pI ~ – theoretical pI; MS/MS score – protein score given by Mascot; % Seq – percentage sequence coverage; Pep match – number of peptides assigned to protein; K test – D value from Kolmogorov-Smirnov test (0.10, D > 0.642); Fold FBS 0%/10 % – fold optical density of the protein spot (expression) for serum depleted proteomes (0 % FBS) compared to control proteomes (10 % FBS); ^a^ – uniquely expressed protein spot. Relevant GO terms for molecular function and biological process are used

Comparing 10 % FBS (control) with 0.5 and 0 % FBS, it could be seen that partial and complete serum depletion induces multiple and pronounced changes in the protein spots. The percentage of differential protein spots was 25 % with 0.5 % FBS (Fig. 2) but 84 % with 0 % FBS (Fig. 4a), of which 40.3 % are protein spots uniquely expressed in the presence of serum, as we expected. The absence of serum had a profound influence on processes such as glycolysis and cell motility (Fig. 4b), as can be deduced from the differential expression of proteins such as PKM1/2, ENO1 and vimentin (Table 1, Additional files 1, 2, 3, 4 and 5).

**Fig. 6:**
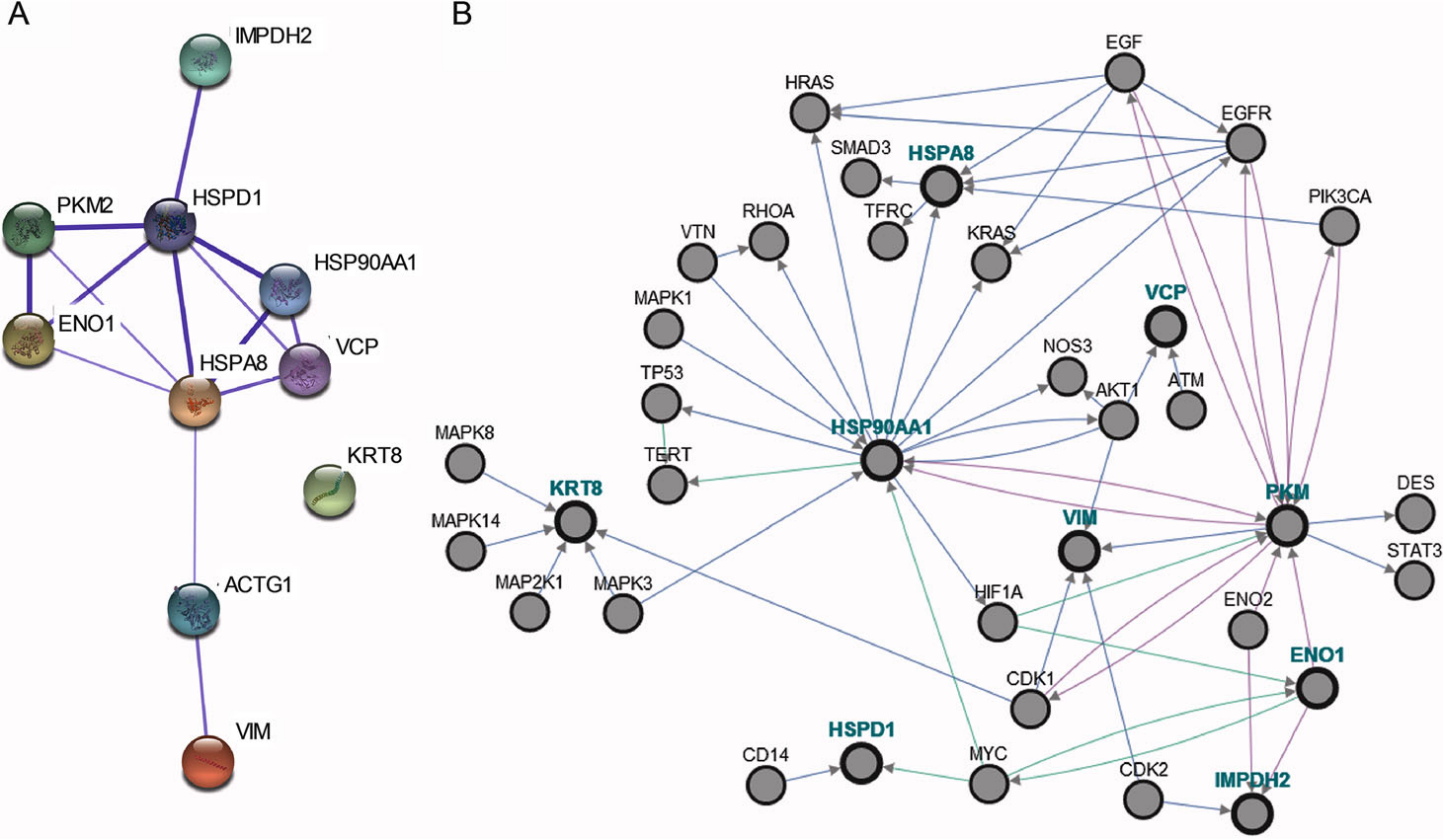
Network analysis of proteins differentially expressed in response to serum depletion. **a** Confidence view of STRING network. Protein association network of differentially expressed identified proteins, powered by STRING software 9.05. (12); a medium confidence score (0.400) and no additional nodes were used as parameters. Thicker lines represent stronger associations. **b** Enriched network generated with PCViz. Protein association network of differentially expressed identified proteins enriched with 36 background nodes generated by PCViz (13). Blue: controls state change, green: controls expression; purple: consecutive catalysis

To gain insight into the predicted physical and functional relationships between the identified proteins showing differential expressions in response to serum depletion, we performed a bioinformatics analysis using the STRING software [17]. A high-confidence network in terms of correlation of interaction data was obtained. The p-value was 5.87 × 10^-12^ and 14 interactions were observed with an interaction expected value (E) of 1.02 for 10 proteins. With the exception of KRT8, all the identified proteins were connected with a central cluster composed of heat shock proteins, PKM2, ENO1 and VCP, as observed in the summary network (Fig. 6a). According to STRING Enrichment, all proteins correspond to placenta tissue (*n* = 10, p-value = 4.019 × 10^-15^, genome background, false discovery rate filter, high confidence).

Cell signaling and metabolic pathways linked with the identified proteins were visualized with PCViz Software [18]. A network of identified proteins enriched with background nodes was built. Figure 6b shows the results of the network analysis, illustrating how different signal transduction pathways are connected and the dependencies on the regulation by serum depletion.

Serum depletion downregulated proteins, such as HSPA8, HSP90AA1, HSPD1 and VCP, are connected with small G proteins, such as Ras and Rho, Akt, and MAPK transduction family members, in concurrence with the mitogenic FBS function and the growth factors that are present and that activate these mitogenic signal pathways.

Serum depletion upregulated proteins, as PKM2, ENO1, IMPDH2, and VIM, are related with cyclin-dependent kinase (CDK). Interestingly, PKM2 interacts with the intermediate filament desmin and signal transducer and activator of transcription STAT3. These two proteins sets are interconnected by hypoxia inducible factor 1 (HIF1A), phosphatidylinositol 3-kinase (PI3K), epidermal growth factor EGF/EGFR and transcription factor Myc (Fig. 6b).

### Vimentin protein identification

**Fig. 7:**
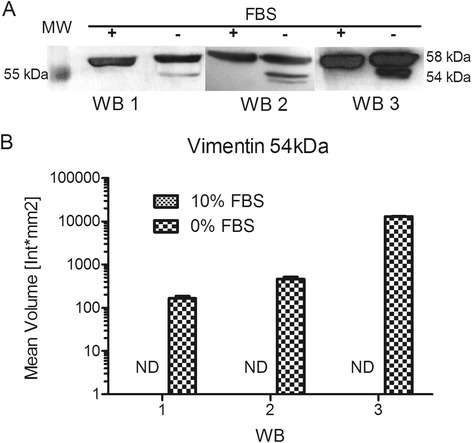
Vimentin western blot analysis. HTR-8/SVneo cells were grown in medium with 10 or 0 % FBS for 24 h. Total protein extracts (30 μg) of independent experimental triplicates were separated by SDS-PAGE. Western blot, as previously described, was used to analyze the protein levels of vimentin. **a** Representative western blot films obtained. MW – molecular weight marker (PageRuler Plus Prestained Protein Ladder, Thermo Scientific). **b** Densitometric quantifications of expected vimentin band (54 kDa) were made in triplicate to each film using Quantity One software. FBS 10 % ND – not detectable

We confirmed our 2DE results via western blot analysis of vimentin, a well-known protein for its key role in phenotype and epithelial–mesenchymal transition (EMT). Evaluation of vimentin protein levels via western blot confirmed the previous changes in expression (upregulation in response to serum depletion; Fig. 7). A sharp band at 54 kDa corresponding to the predicted molecular weight for the protein was detected only in serum-depleted cells.

## Discussion

In this study, we found that complete serum depletion and culture with only 0.5 % serum differently affect the protein expression of HTR-8/SVneo immortalized trophoblast cells. The identified proteins include cytoskeletal proteins, metabolic enzymes and heat shock chaperones. We also predicted the interactions between them using the STRING and PCViz networks, which all together correlated with differential growth rates depending on the complete or partial depletion.

This exploratory proteomic analysis proves that there is a deep change in cells in response to serum depletion, which is a common but careless practice in cell culture, and offers new target proteins for further study. However, the in vivo effects of serum depletion on the related biological process must be further studied.

The percentage of differentially expressed proteins in response to serum depletion was larger than those expressed in response to 0.5 %, which is usually considered a low serum dose. Furthermore, key proteins related with metabolism – PKM1/2 and ENO1 – and vimentin were found to be differentially expressed under conditions of complete serum depletion but not in 0.5 % FBS culture. These results indicate additional phenomena that may include a reprogramming of the transcriptional process in response to the microenvironmental conditions.

VIM is an intermediate filament with transcription that is highly regulated by serum and used as a mesenchymal marker, whereas KRT8 is a marker of epithelial cells [19]. Changes in these proteins correlate with EMT: VIM is upregulated while KRT8 is downregulated in cells undergoing EMT [20]. Accumulating evidence suggests a critical role of EMT in cancer progression [21, 22]. Interestingly, our results show a similar behavior for these proteins in response to serum depletion in trophoblasts. An important body of evidence gives support to the comparable behavior of normal trophoblast cells and malignant cells [2]. Although trophoblast cells acquire some mesenchymal characteristics, such as VIM expression, they retain their epithelial phenotype, with characteristics including growing in monolayer and expressing KRT8.

Serum depletion induced the expression of the enzymes ENO1, PKM2 and IMPDH2. ENO1 is a multifunctional enzyme that, besides its role in glycolysis, takes part in several processes such as growth control, hypoxia tolerance and transcription repression through binding to the *c-myc* promoter [23]. PKM2 has different roles beyond rate-limiting control in glycolysis. PKM2, the embryonic/tumor splicing isoform, participates in a positive feedback loop that promotes HIF-1 transactivation and reprogramming of glucose metabolism in cancer cells, thus contributing to tumorigenesis [24].

In recent years, research on the link between altered metabolism and tumor growth promotion has accelerated [25]. Via the known Warburg effect, cancer cells exhibit a high rate of glycolysis and glucose uptake, even in the presence of oxygen [26]. Van der Heiden et al. argued that the major function of aerobic glycolysis is to maintain high levels of glycolytic intermediates to support anabolic reactions in cells [27], and has described how growth factor signaling induces uptake of nutrients such as glucose and glutamine that fuel bioenergetics and biosynthetic cellular pathways [28]. Also, through phosphoproteomic approaches, it was found that tyrosine phosphorylation regulates PKM2 activity to provide a metabolic advantage to tumor cells, thereby promoting tumor growth [29]. In reference to our results with serum-depleted culture, restricting exogenous growth factors could induce alterations in cellular signaling that promote the Warburg effect and a protein profile related to tumor growth, linking signal transduction with metabolism.

IMPDH2 is a rate-limiting enzyme for de novo synthesis of guanine nucleotides and therefore involved in the regulation of cell growth. It is plausible to consider a role for this enzyme in the development of malignancy and the progression of some tumors [30, 31].

These findings suggest that serum depletion could promote a switch in the trophoblast protein expression that matches with malignant cell skills. Trophoblast cells share several common features with malignant cells, but trophoblast processes are narrowly controlled. Several studies support the hypothesis that trophoblast and cancer cells use similar mechanisms implemented by identical molecular circuits to achieve their processes [2, 32]. This fact, together with our results, supports the proposed use of trophoblast cells as a biological model to understand the malignant process and its signal transduction activation.

It is known that at early stages of development, solid tumors are often exposed to environmental stressors such as hypoxia, acidosis and nutrition deprivation. In general, tumor cells overcome these conditions and develop into more malignant phenotypes [33, 34]. In vivo, extrinsic factors, such as poor tumor vascularization generate a stressful environment that alters gene expression and the proteome. The main changes include: increased transcription of cytokines, chemokines and growth factors; elevated activity of DNA-repair enzymes, proteases and other degradative enzymes and resistance-related proteins; and changes in adhesion molecules and components of the innate and adaptive immunity [35]. Therefore, the pathophysiologic environment of tumors has been linked to a more aggressive phenotype, playing a role in tumor progression and metastatic disease [36]. During periods of metabolic stress and growth factor restriction, cancer cells use autophagy as a strategy to sustain metabolism [37] and SV40 ST antigen in transformed cancer cells could induce autophagy as an alternate energy source in stress conditions [38].

The intrauterine environment during the first weeks of gestation resembles this tumor microenvironment, modulating the placental development and trophoblast function. It takes place in low oxygen conditions, meaning that the metabolism is probably largely anaerobic during the period of organogenesis. It is supported by secretions from the endometrial glands [39, 40], such as several cytokines and growth factors, where conceptus, trophoblast and endometrial glands establish a paracrine and/or autocrine signaling network [1, 2, 32]. A limited trophoblast invasion of maternal spiral arteries correlates to both preeclampsia and fetal growth restriction [41]. Other results show the implications of hypoxia on trophoblast function with a dual effect according to the gestation week: low metabolism and oxygen concentration levels during the initial development of the blastocyst will benefit the pregnancy outcome [40, 42], whereas hypoxia inhibits trophoblast differentiation and invasion and has implications in trophoblast apoptosis after the 7th week of gestation [1, 43]. However, the effects of null or low serum doses, seen as nutrition and growth factor restriction, have been poorly understood.

Proteomic studies have been accomplished on trophoblast cells, examining the proteome changes during BeWo choriocarcinoma cells differentiation [44] and syncytialization [45], but there had thus far been no studies about serum depletion. However, a proteomic analysis of hypoxia-induced responses on the BeWo choriocarcinoma cell line showed that two proteins involved in the glycolytic pathway (malate dehydrogenase and enolase) were upregulated, while the expression of two cytoskeletal components (keratin 1 and beta-actin) were downregulated [45]. Interestingly, whereas glycolysis is stimulated in response to hypoxia, in our study this pathway is stimulated by serum depletion, reinforcing the idea of a similar regulation by hypoxia and serum depletion.

Null or low serum doses could behave as a stressor that may influence normal placental growth and development in the early stages of pregnancy. Here, FBS is used to resemble physiological conditions for the in vitro culture, because it contains nutrients along with plenty of growth factors and hormones. Despite its bovine origin, these have a proliferative and invasion-stimulating effect on HTR-8/SVneo cells [9, 12].

In addition, HTR-8/SVneo cells secrete several growth factors and hormones including hCG. After 24 h of culture, the medium is conditioned with these soluble factors, generating a sustained autocrine signaling [7, 9].

Although in vitro cell culture experiments and treatments are done in absence of serum, few studies have focused on the influence of serum depletion on cellular response [3], but recent results do suggest that it is not simply mediated by growth arrest but controlled by unknown regulatory proteins [46]. A synergistic effect between serum and insulin or insulin-like growth factor-I (IGF-I) is seen in choriocarcinoma cells [47]. In other biological models, such as human placental decidua basalis (termed as PDB-MSCs; a type of stem cell), cells are resistant to hypoxia and serum deprivation [48]. Regarding HTR-8/SVneo cell line, a study showed common features with stem cells, “probably attained through corruption of ‘stemness-’ associated with transcription factor networks”, indicating a certain degree of plasticity [49] and reinforcing our hypothesis of an adaptive switch, similar to malignant cells.

There are few in vivo biological states in which serum levels are minimal. Examples include isquemia [50, 51], aestivation and metabolic depression [52]. However, treatments in the absence of serum are a common cultural practice in research, but there is a need to consider the inherent effects of serum depletion. In this study, we have demonstrated relevant proteomic changes in response to serum depletion, and these could be part of a survival strategy aligned with the physiological role of extravillious trophoblast cells. Therefore, in those cases where a lower FBS dose in indicated, the use of 0.5 % doses instead of complete depletion is recommended.

Similarly, just as the tumor microenvironment may have an influence on the tumor cell function and development, the extreme intrauterine environment (modeled here as complete serum depletion) induces an adaptive response in trophoblast cells, involving EMT and a flexible metabolic adjustment increasing glycolysis. In this study, we identified differential protein expression changes in response to different serum doses, using 2DE and mass spectrometry. The identified proteins are the first evidence of a protein expression response to different serum doses in an immortalized trophoblast-derived cell line.

## Conclusions

In conclusion, our results suggest a phenotype change related to malignant progression, in response to serum depletion on a first trimester human trophoblast model. However, additional studies are needed to determine if these changes are due to the lack of nutrients or signaling molecules.

## Additional files

Additional file 1: **Table S2.** Preliminary assay: proteins identified via MS and bioinformatics analysis from 0.5 % serum culture. HTR-8/SVneo cells were grown in medium with 10 or 0.5 % FBS for 24 h. Total protein extracts (0.15 mg) were separated by 7 cm 2DE gels as previously described. Protein spots were analyzed via MALDI TOF and identified using the MASCOT search engine. MW (kDa) – theoretical and experimental molecular weight in kDalton; pI ~ – theoretical pI; MS score – protein score given by Mascot; % Seq – percentage sequence coverage; Pep match – number of peptides assigned to protein; Fold FBS 0.5 %/10 % – fold optical density of protein spot (expression) of 0.5 % serum proteomes (0.5 % FBS) compared to control proteomes (10 % FBS). Relevant GO terms for molecular function and biological process, retrieved by Nextprot database and DAVID tool. (XLS 57 kb)

Additional file 2: **Table S3.** DIGE analysis: proteins identified via MS and bioinformatics analysis from 0.5 % serum culture. HTR-8/SVneo cells were grown in medium with 10 or 0.5 % FBS for 24 h. Total protein extracts (40 μg) were labeled with either Cy2 or Cy3 and analyzed using 2D–DIGE technology in triplicate, as previously described. Protein spots were analyzed via MALDI TOF and identified using the MASCOT search engine. MW (kDa) – theoretical and experimental molecular weight in kDalton; pI ~ – theoretical pI; MS score – protein score given by Mascot; % Seq – percentage sequence coverage; Pep match – number of peptides assigned to protein; Fold FBS 0.5 %/10 % – fold optical density of protein spot (expression) of 0.5 % serum proteomes (0.5 % FBS) compared to control proteomes (10 % FBS). Relevant GO terms for molecular function and biological process, retrieved by Nextprot database and DAVID tool. (XLS 90 kb)

Additional file 3: Vimentin, citokeratin 8, pyruvate kinase and enolase 1 differential expression due to lack of serum. Magnified gel images of representative differentially expressed protein spots on 2DE gels, comparing HTR8/SVneo culture containing 0 or 10 % FBS. Images on the left correspond to 10 % FBS and those on the right to 0 % FBS. Vimentin (Vim), citokeratin 8 (KRT8), pyruvate kinase (PKM1/2) and enolase 1 (ENO1) were found to be differentially expressed. (TIF 4551 kb)

Additional file 4: Vimentin, pyruvate kinase and enolase 1 expression remain invariant in 0.5 % FBS proteomes. Magnified gel images of representative protein spots on DIGE gels, comparing HTR8/SVneo culture containing 0.5 or 10 % FBS. Images on the left correspond to 10 % FBS and those on the right to 0.5 % FBS. Pyruvate kinase (PKM1/2) was identified in four protein spots, and enolase 1 (ENO1) was identified in two protein spots, each one invariable. (TIF 2836 kb)

Additional file 5: Table S4. Database accession of proteins identified via MS/MS and bioinformatics analysis. (XLS 106 kb)

## Abbreviations

2DE: Two-dimensional gel electrophoresis
DIGE: Fluorescence difference gel electrophoresis
DTT: Dithiothreitol
EMT: Epithelial-mesenchymal transition
ENO1: Alpha enolase
ESI: Electrospray ionization
FBS: Fetal bovine serum
GO: Gene ontology
IEF: Isoelectric focusing
IMPDH2: Inosin-5’-monophosphate dehydrogenase 2
KRT8: Cytokeratine 8
LC: Liquid chromatography
MALDI: Matrix-assisted laser desorption/ionization
MS: Mass spectrometry
MS/MS: Mass spectrometry in tandem
PAGE: Polyacrylamide gel electrophoresis
PKM1/2: M1/M2 pyruvate kinase
SDS: Sodium dodecyl sulfate
TOF: Time of flight mass analyzer
Vim: Vimentin
