# Supplementary figures and images for: Serum depletion induces changes in protein expression in the trophoblast-derived cell line HTR-8/SVneo
Source: Cell Mol Biol Lett. 2016 Oct 16;21:22. doi: 10.1186/s11658-016-0018-9 (PMC5415790; doi:10.1186/s11658-016-0018-9)

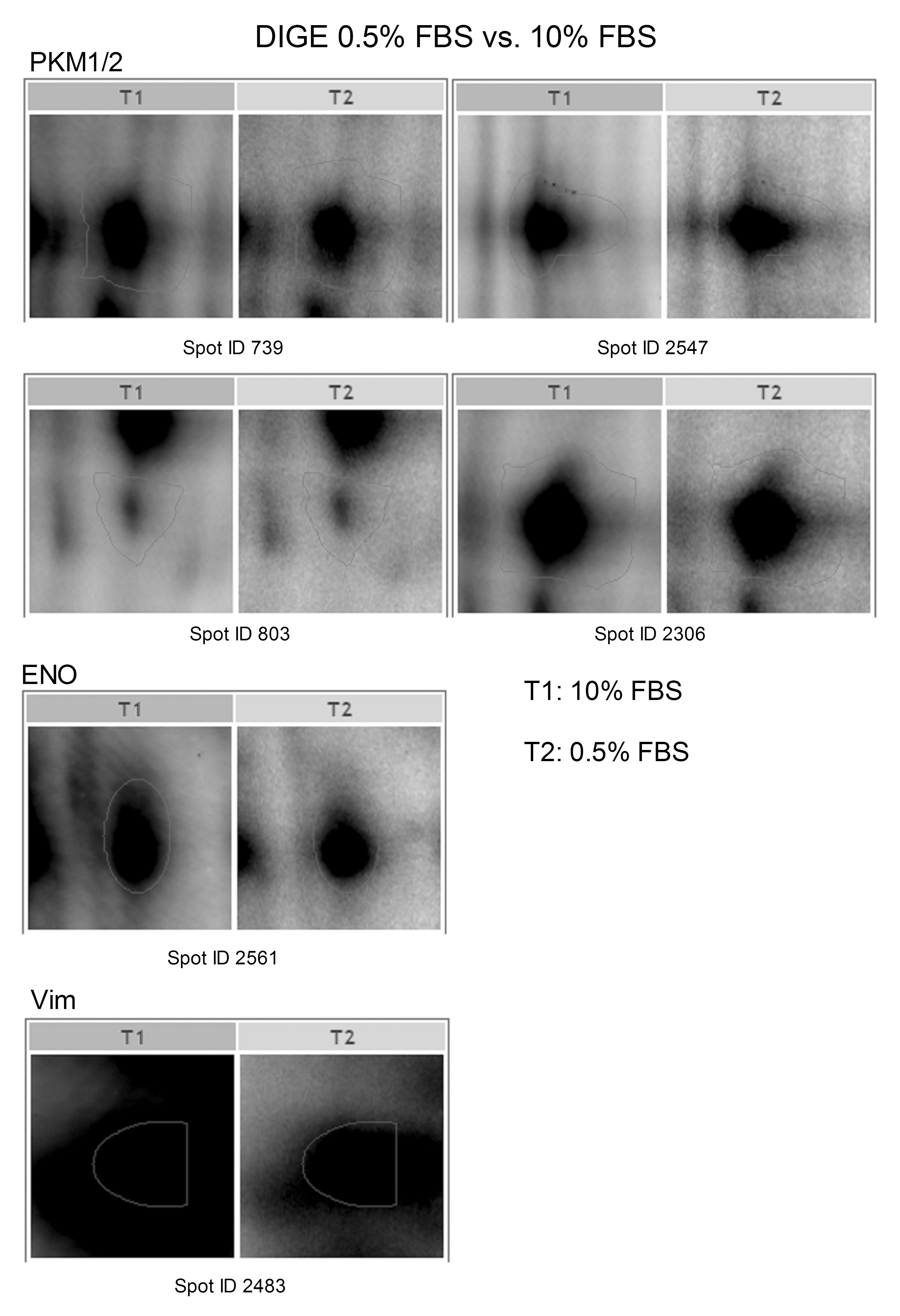

Supplement: Supplementary file 3 — Vimentin, citokeratin 8, pyruvate kinase and enolase 1 differential expression due to lack of serum. Magnified gel images of representative differentially expressed protein spots on 2DE gels, comparing HTR8/SVneo culture containing 0 or 10 % FBS. Images on the left correspond to 10 % FBS and those on the right to 0 % FBS. Vimentin (Vim), citokeratin 8 (KRT8), pyruvate kinase (PKM1/2) and enolase 1 (ENO1) were found to be differentially expressed. (TIF 4551 kb) [file 11658_2016_18_MOESM3_ESM.tif]

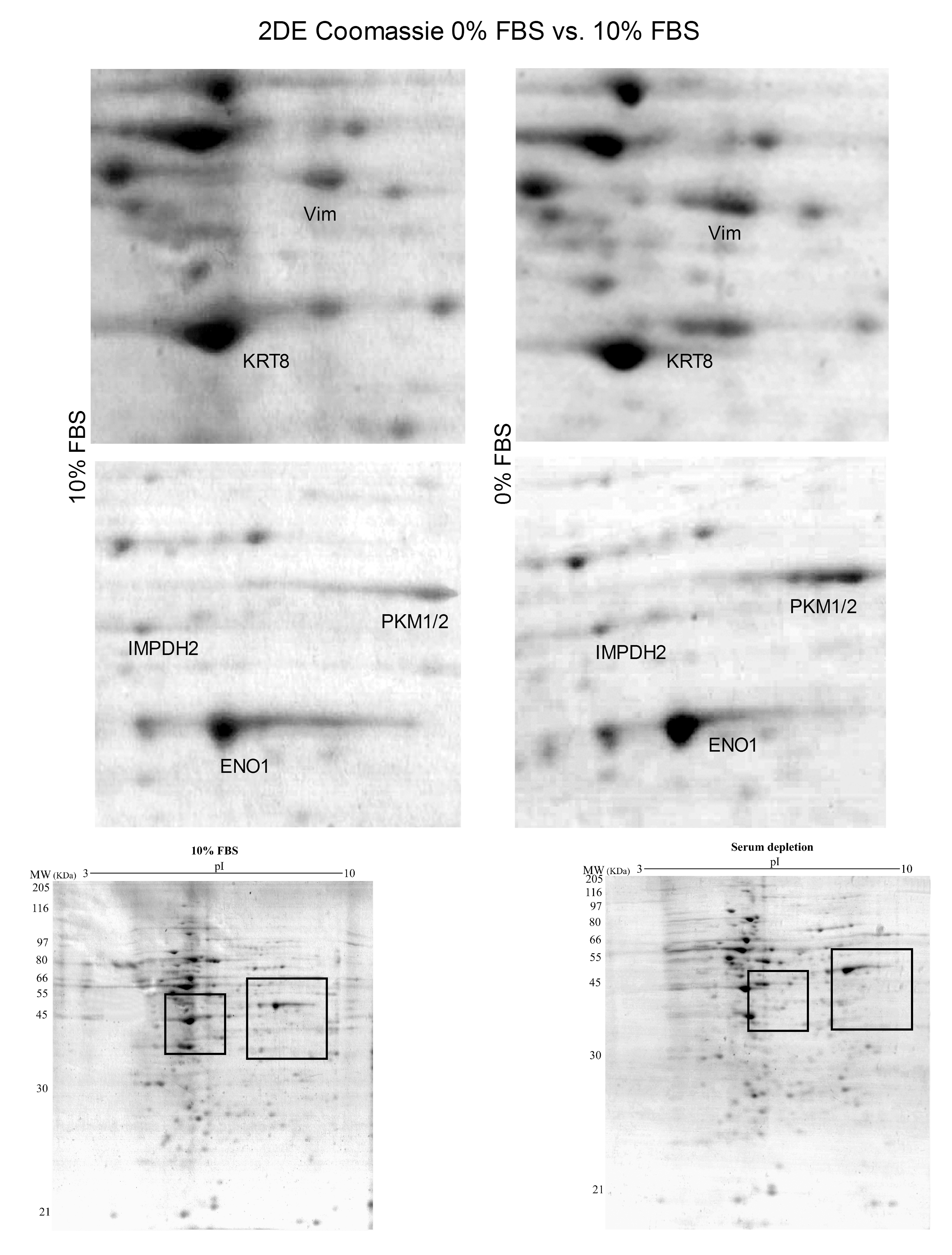

Supplement: Supplementary file 4 — Vimentin, pyruvate kinase and enolase 1 expression remain invariant in 0.5 % FBS proteomes. Magnified gel images of representative protein spots on DIGE gels, comparing HTR8/SVneo culture containing 0.5 or 10 % FBS. Images on the left correspond to 10 % FBS and those on the right to 0.5 % FBS. Pyruvate kinase (PKM1/2) was identified in four protein spots, and enolase 1 (ENO1) was identified in two protein spots, each one invariable. (TIF 2836 kb) [file 11658_2016_18_MOESM4_ESM.tif]
